# A genome-wide association study for prolificacy in three Polish sheep breeds

**DOI:** 10.1007/s13353-021-00615-6

**Published:** 2021-02-20

**Authors:** Grzegorz Smołucha, Artur Gurgul, Igor Jasielczuk, Aldona Kawęcka, Anna Miksza-Cybulska

**Affiliations:** 1 Department of Animal Molecular Biology, National Research Institute of Animal Production, Krakowska 1, 32-083 Balice, Poland; 2 Center for Experimental and Innovative Medicine, University of Agriculture in Krakow, Rędzina 1c, 30-248 Kraków, Poland; 3 Department of Sheep and Goat Breeding, National Research Institute of Animal Production, 32-083 Krakowska 1, 32-083 Balice, Poland

**Keywords:** GWAS, Sheep, EPHA6, Prolificacy, SNP

## Abstract

Reproductive traits (especially litter size) are usually characterized by low heritability, and thus, phenotypic selection is often ineffective and slow. In order to improve fertility characteristics such as ovulation rate and litter size, it seems more effective to select breeding animals based on their genotype. The aim of the study was to use genome-wide association study (GWAS) in three sheep breeds to identify the genetic variants affecting the litter size in sheep. The study allowed us to identify one genome-wide significant SNP (rs402032081—located in ephrin type-A receptor 6, *EPHA6*) showing an association with litter size in Polish Mountain Sheep. We suggest that the *EPHA6* gene can be a candidate gene for prolificacy trait in selected breeds of sheep; however, it needs further functional data for validation.

**Supplementary Information:**

The online version contains supplementary material available at 10.1007/s13353-021-00615-6.

## Introduction

Modern technology in biology and in molecular genetics allowed to identify genes and polymorphisms that are responsible for improving functional traits important for farm animal production (Smołucha et al. 2020). In sheep (Ovis aries), searching for candidate genes responsible for the production trait focuses mainly on reproductive traits (Davis 2004; Abdoli et al. 2016). The reproductive ability could be measured by fertility, fecundity, and prolificacy. Fertility is defined as a number of lambing per year, fecundity is the number of lambs produced per year, and prolificacy is defined as a litter size. Several studies using genome-wide association study (GWAS) successfully identified genetic variants associated with the prolific phenotype in French Grivette and Polish Olkuska sheep breed populations near a functional candidate gene on the X chromosome (Demars et al. 2013). A study conducted by Cockrum et al. (2012) on Blackface sheep breed showed 10 SNPs that reach the nominal genome-wide d threshold for birth type, but only 4 candidate genes were identified: *ODZ1* and *ODZ3*, *LTBP3*, and *DSCAM* as a potential gene with impact on fertility traits. The same author identified genes from the ephrin family which shows a significant correlation with backfat (Ephrin type B receptor 1, 2, 3, Ephrin type-A receptor 2, 3, 7) (Cockrum et al. 2012). In different research conducted by Xu et al. (2018) using five sheep breeds with high prolificacy (Wadi, HU, Icelandic, Finnisheep Romanov, and one with low prolificacy—Texel), the authors identified different sets of candidate genes associated with litter size in different breeds: *BMPR1B*, *FBN1*, and *MMP2* in Wadi; *GRIA2*, *SMAD1*, and *CTNNB1* in Hu; *NCOA1* in Icelandic; *INHBB*, *NF1*, *FLT1*, *PTGS2*, and *PLCB3* in Finnsheep; and *ESR2* in Romanov and *ESR1*, *GHR*, *ETS1*, *MMP15*, *FLI1*, and *SPP1* in Texel (Xu et al. 2018). Benavides et al. (2018) in a population in which segregates a major gene determinant of prolificacy in sheep—Vacaria-identified variants showing association on sheep chromosome 5 (43,415,384–43,708,878 bp). Three significant markers were in linkage disequilibrium with OAR5_45481559, a marker within the *GDF9* gene that showed nominal *p* value significance (Benavides et al. 2018). The aim of this study was to use GWAS to identify SNPs affecting the prolificacy traits in three mountain sheep breeds (Colorful Mountain Sheep (CMS), Polish Mountain Sheep (PMS), and Podhale Zakelska Sheep (PZ)) which represents the native Polish sheep population. These breeds are perfectly adapted to the difficult local environment, with changing climatic conditions and a short growing season. It is noteworthy that the mountain sheep are practically the only milk sheep in the country. Moreover, sheep included in the genetic resources conservation program constitute a valuable element of the genetic diversity of this species and play an important role in the local ecosystems (Kawęcka et al. 2020).

## Material and methods

Blood samples obtained from 155 randomly selected female sheep belonging to three native Polish breeds were analyzed. Animal procedures were approved by the Local Animal Care Ethics Committee No. II in Kraków - permission number 1293/2016 in accordance with EU regulations. The breeds included in the study included Podhale Zackel (PZ, *n*= 74), Polish Mountain Sheep (PMS, *n*=36), and Colored Mountain Sheep (CMS, *n*=45). For each sheep, prolificacy data was recorded as the number of lambs per litter (litter size). The average fertility is calculated on the basis of based on the first three litters for each sheep. DNA was purified using the QuickGene DNA Whole Blood Kit (Kurabo). The obtained DNA was quality controlled and analyzed with the use of OvineSNP50 BeadChip (Illumina, San Diego, CA, USA) which targets 54,241 SNPs, following standard Infinium Ultra Protocol. The obtained genotypes were controlled for quality by evaluation of CallRate, and only samples with values >95% were further analyzed. The individual SNPs have also been filtered across the population, and only SNPs with minor allele frequency (MAF) >0.01, missing genotypes <20%, and not deviating from Hardy-Weinberg Equilibrium (*p*>0.0001) have been analyzed. Additionally, SNPs without a known position in the Oar_v3.1 genome build have been removed.

Genome-wide association analysis of SNP genotypes with fertility data was performed for combined breeds set using linear regression implemented in the Plink software (Purcell et al. 2007). To account for population stratification, the first two principal components have been used as a covariate in this analysis. PC analysis was performed on the pruned dataset, in which one of the variants with *r*^2^>0.5 in the 50-SNP window has been excluded. Additionally, linear regression was performed in each breed separately. The obtained *p* values have been corrected for multiple testing by the FDR procedure (Benjamini and Hochberg 1995). The genomic region around a significant SNP was screened for genes using the UCSC Genome Browser.

## Results and discussion

**Fig. 1:**
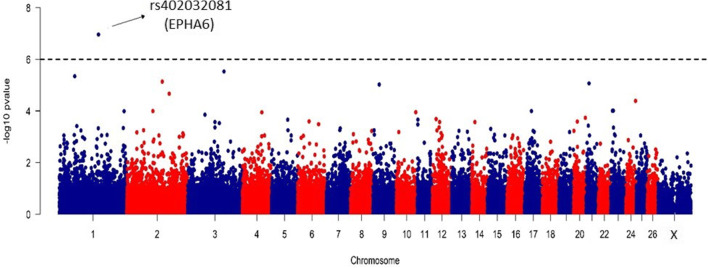
Manhattan plot for GWAS on fertility traits in Polish Mountain Sheep (PMS)

The association analysis was performed on a final filtered set of 49,204 SNPs with an average inter-marker distance of 52.38 kb and mean MAF ranging from 0.277 (±0.138) to 0.290 (±0.131) in PMS and PZ, respectively. The average observed heterozygosity for the filtered SNPs raged from 0.373 in PMS to 0.383 in PZ. The average prolificacy for the studied breeds was the highest for CMS (1.369 ±0.23) and the lowest for PMS (1.2 ±0.29). The performed linear regression for all breeds did not result in any significant SNPs after correction for multiple testing (Supplementary File 2). The analysis performed for each breed separately (Supplementary File 3) showed a clear association (FDR=0.005) of SNP—OAR1_172690647.1, localized on chromosome 1 (160,114,886 bp, rs402032081) with fertility traits only in Polish Mountain Sheep (Fig. 1; Table 1). This SNP was located in the *EPHA6* (ephrin type-A receptor 6) gene and in the vicinity of the U6 spliceosomal RNA (ENSOART00000025351) gene and other provisional ENSEMBL genes (ENSOART00000018925).

**Table 1:** Results of the association analysis for the single detected significant SNP on OAR1

CHR	SNP	Position	Tested allele	Regression coefficient	Coefficient *t*-statistic	Asymptotic *p* value for *t*-statistic	FDR
1	OAR1_172690647.1	160,114,886	A	0.5625	6.693	1.102e−07	0.0053

*CHR* chromosome, *SNP* SNP name, *FDR* false discovery rate

The analyzed sheep breeds are characterized by relatively low prolificacy when compared to, e.g., highly proliferative breeds like Olkuska or Romanov sheep, and thus, no variants associated with high prolificacy have been expected. Nevertheless, while considering existing genetic variation in the studied sheep breeds for this trait, other genes affecting prolificacy could be detected in the analyzed breeds. Our GWAS analysis identified one genome-wide significant SNP (rs402032081—located in ephrin type-A receptor 6, *EPHA6*) showing an association with litter size in Polish Mountain Sheep. *EPHA6* in mouse embryos is highly expressed during the development of the central nervous system, in adult animals in the hypothalamus, thalamus, and amygdala (dos Santos et al. 2017). GWAS analysis performed in cattle connect polymorphism in *EPHA6* with temperament phenotypes, i.e., reactivity, anxiety, and aggression (dos Santos et al. 2017). *EPHA6* is related to the MAPK/ERK pathway (also known as the Ras-Raf-MEK-ERK pathway) and Development Slit-Robo signaling (https://www.genecards.org/cgi-bin/carddisp.pl?gene=EPHA6). Both these pathways have great potential to regulate early development and cell proliferation and thus affect prolificacy. The ERK cascade is activated by a variety of extracellular agents, including growth factors, hormones, and also cellular stresses to induce cellular processes that include mainly proliferation and differentiation, so the important processes for developing an embryo (McCain 2013). Slit-Robo signaling instead is best known for mediating axon repulsion in the developing nervous system; however, in recent years, the functional repertoire of Slits and Robo has expanded tremendously and has been linked to roles in cell proliferation, stem cell regulation, angiogenesis, and organ development, as well as to tumorigenesis and other diseases (Blockus and Chédotal 2016). Regulation of both these pathways has the potential to affect embryo development and thus *EPHA6* can be a good candidate for prolificacy trait in sheep. Unfortunately, our GWAS has been based on relatively small sample sizes, and unfortunately, this work is limited by the number of genotyped animals. The low heritability of the trait and small sample sizes hinder the detection of strong and reliable association signals and only suggest that *EPHA6* is a candidate gene for prolificacy that can be formulated which definitely requires additional data for validation.

## Supplementary Information

Supplementary File 1. Principal component analysis for the studied sheep breeds. (PNG 11 kb)

Supplementary File 2. Results of GWAS across breeds. (XLSX 5181 kb)

Supplementary File 3. Results of GWAS within separate breeds. (XLSX 14986 kb)
