# Supplementary figures and images for: A genome-wide association study for prolificacy in three Polish sheep breeds
Source: J Appl Genet. 2021 Feb 20;62(2):323–6. doi: 10.1007/s13353-021-00615-6 (PMC8032615; doi:10.1007/s13353-021-00615-6)

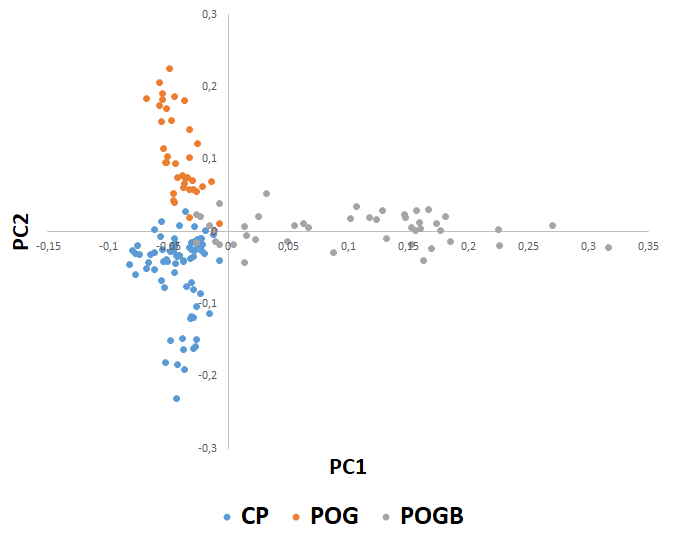

Supplement: Supplementary file 1 — Principal component analysis for the studied sheep breeds. (PNG 11 kb) [file 13353_2021_615_MOESM1_ESM.png]
